# Flavonoid Derivative of *Cannabis* Demonstrates Therapeutic Potential in Preclinical Models of Metastatic Pancreatic Cancer

**DOI:** 10.3389/fonc.2019.00660

**Published:** 2019-07-23

**Authors:** Michele Moreau, Udoka Ibeh, Kaylie Decosmo, Noella Bih, Sayeda Yasmin-Karim, Ngeh Toyang, Henry Lowe, Wilfred Ngwa

**Affiliations:** ^1^Brigham and Women's Hospital, Dana-Farber Cancer Institute, Harvard Medical School, Boston, MA, United States; ^2^Department of Physics, University of Massachusetts Lowell, Lowell, MA, United States; ^3^Department of Biology, University of Massachusetts Boston, Boston, MA, United States; ^4^Department of CaNCURE Program, Northeastern University, Boston, MA, United States; ^5^Flavocure Biotech Inc., Baltimore, MD, United States

**Keywords:** pancreatic cancer, flavonoids, cannabis, metastasis, radiotherapy, smart biomaterials

## Abstract

Pancreatic cancer is particularly refractory to modern therapies, with a 5-year survival rate for patients at a dismal 8%. One of the significant barriers to effective treatment is the immunosuppressive pancreatic tumor microenvironment and development of resistance to treatment. New treatment options to increase both the survival and quality of life of patients are urgently needed. This study reports on a new non-cannabinoid, non-psychoactive derivative of cannabis, termed FBL-03G, with the potential to treat pancreatic cancer. *In vitro* results show major increase in apoptosis and consequential decrease in survival for two pancreatic cancer models- Panc-02 and KPC pancreatic cancer cells treated with varying concentrations of FBL-03G and radiotherapy. Meanwhile, *in vivo* results demonstrate therapeutic efficacy in delaying both local and metastatic tumor progression in animal models with pancreatic cancer when using FBL-03G sustainably delivered from smart radiotherapy biomaterials. Repeated experiments also showed significant (*P* < 0.0001) increase in survival for animals with pancreatic cancer compared to control cohorts. The findings demonstrate the potential for this new cannabis derivative in the treatment of both localized and advanced pancreatic cancer, providing impetus for further studies toward clinical translation.

## Introduction

Pancreatic ductal adenocarcinoma is an antagonistic internecine ailment of the exocrine pancreas with < 8% of patients surviving within a 5-year period (1, 2). A major challenge shared by pancreatic cancers is its aggressiveness, which often metastasizes to other organs before the patient is even diagnosed (3, 4).

Current treatment options for pancreatic cancer include: surgery, chemotherapy, targeted therapy, immunotherapy, and radiation therapy. Curative treatment is available only if the tumor is found early and can be removed by surgery before metastasis. If the cancer has metastasized, the standard of care is chemotherapy, or radiotherapy. However, pancreatic cancer is notoriously defiant to current therapies including chemotherapy, radiotherapy and immunotherapy (1, 5).

Cannabinoids, which are the bioactive components of *Cannabis sativa* and their derivatives, have been investigated as both anti-cancer agents and for managing the side effects of conventional cancer treatments like chemotherapy and radiotherapy (6). Previous studies have indicated that medical cannabis derivatives could enhance survival in pancreatic cancer animal models, when used in synergy with radiotherapy (7). Smart materials for drug delivery like the smart radiotherapy biomaterials (SRBs) system have also been investigated for delivering cannabinoids into tumors to enhance radiotherapy treatment for pancreatic cancer (8). A major benefit of the SRB approach is their ability to be employed in place of currently used inert radiotherapy biomaterials (e.g., spacers, or fiducial markers) and hence their use could come at no additional inconvenience to patients.

In this study, we investigate a new non-cannabinoid, non-psychoactive derivative of cannabis, called FBL-03G, to assess its potential for the treatment of pancreatic cancer. We hypothesize that the use of FBL-03G will have therapeutic potential and can enhance radiotherapy during the treatment of pancreatic cancer. To investigate this hypothesis, *in vitro* studies were first carried out with and without radiotherapy (RT). *In vitro* studies, *in vivo* studies were also conducted in small animals employing FBL-03G sustainably delivered from smart radiotherapy biomaterials, allowing continual exposure of the tumor to the cannabis derivative payloads over time.

Apart from the antineoplastic properties of cannabis derivatives, the immune system modulative properties of these extracts have been well documented (8–12). Different volumes and concentrations of FBL-03G payloads were also investigated for their potential to generate systemic tumor responses. In particular, we investigated the abscopal effect, whereby radiotherapy (RT) at one site may lead to regression of metastatic cancer at distant sites that are not irradiated (13). The abscopal effect has been connected to mechanisms involving the immune system (14). However, the abscopal effect is rare because at the time of treatment, established immune-tolerance mechanisms may hamper the development of sufficiently robust abscopal responses. Today, the growing consensus is that combining radiotherapy with immunoadjuvants provides an opportunity to boost abscopal response rates, extending the use of radiotherapy to treatment of both local and metastatic disease (15). With in this context, the cannabis derivative FBL-03G was also investigated as a potential immunoadjuvant to radiotherapy.

## Materials and Methods

### Materials and Antibody

Acetone, Dimethyl sulfoxide (DMSO), Poly (lactic-co-glycolic) acid (PLGA) (M.W.: 50–50 kDa), and Crystal Violet dye were acquired from Sigma-Aldrich. The Harvard apparatus was obtained from Harvard Bioscience (Holliston, MA, USA), and silicone tubing (ID 1/32″) was purchased from Saint-Gobain Performance Plastics Laboratory Division (USA). Brachytherapy needles were purchased from IZI Medical Products (MD, USA). All cell culture products (DMEM, RPMI, Trypsin, Fetal Bovine Serum, MEM non-essential amino acids, sodium pyruvate, β-mercaptoethanol, penicillin/streptomycin, and PBS pH 7.4) were obtained from Gibco, Thermo Fisher, and Life Technologies (Waltham, MA, USA). Flavocure Biotech Inc. (Baltimore, MD, USA) supplied the test molecule, FBL-03G with a purity of 98.7% determined by High Performance Liquid Chromatography (HPLC).

### FBL-03G Synthesis

FBL-03G, a flavonoid derived from *Cannabis sativa* L., is the unnatural isomer of Cannflavin B, a metabolite of Cannabis. Through a bioactivity guided isolation approach, 11 flavonoids were isolated using flash chromatography and characterized by nuclear magnetic resonance (NMR) and mass spectrometry (MS) methods (2, 16, 17). Generated spectroscopic data for FBL-03G were similar to those of the following 11 previously isolated and characterized compounds of the Cannabis plant; apigenin (1), Chrysoeriol (2), kaempferol (3), luteolin (4), quercetin (5), vitexin (6), isovitexin (7), orientin (8) and prenylated flavonoids including Cannflavin A (9), Cannflavin B (10) and Cannflavin C (11) (Figure S1) (16–21). The molecules were further screened for kinase inhibition, and chrysoeriol (Cresorol) demonstrated significant activity against FLT3, FLT3-ITD, and FLT3-D835Y and moderate activity against CSF1R (2). FBL-03G demonstrated significant activity against CSF1R kinase and moderate activity against FLT3, FLT3-ITD, FLT3-D835Y, CK2a, CK2a2, Aurora A, Aurora B, Aurora C, and Pim2 (2).

### Fabrication of Smart Radiotherapy Biomaterials (SRB)

This study used combination treatment of FBL-03G (Mw = 368.38 g/mol) as an immunoadjuvant, delivered from smart radiotherapy biomaterial (SRB), and radiotherapy (RT). SRBs were developed following previously reported procedures for fabricating and loading drugs into SRBs. Briefly, 300 mg of Poly (lactic-co-glycolic) acid (PLGA) polymer added to 3.5 mL of acetone was homogenously mixed into a hydrogel (8, 22). The Harvard apparatus was used to provide a constant flow rate of the mixture into the silicon tubing with inner diameter of 1/32′. The PLGA hydrogel loaded in silicon tubing was allowed to cure under 50°C for 48 h. After curing, the silicon tubing was cut to 5 mm length and the SRBs were extracted. Three different concentrations of FBL-03G, respectively, were used as payloads in the SRBs. A small animal radiation research platform (SARRP, Xtrahl, Inc., Suwanee, GA, USA) was used for radiotherapy using 220 kVp, 13 mA, (10 × 10) mm nozzle, and 0.15 mm copper (Cu) filter to deliver 6 gray (Gy) dose. In addition, computed tomography (CT) images of the mice were taken using at 65-kVp and 0.8-mA. Mice were anesthetized with isoflurane and image-guided radiotherapy was used to specifically irradiate tumors on one site as needed.

### Cell Culture

Pancreatic cancer cell line, Panc-02, was obtained from the National Cancer Institute and cultured with Dulbecco's Modified Eagle's Medium (DMEM) with 10% FBS and 1% penicillin/streptomycin. Another pancreatic cancer cell line, Ptf1/p48-Cre (KPC) cells were a gift from Dr. Anirban Maitra (MD Anderson Cancer center). KPC cell line was derived from an LSL-Kras; p53+/floxed, Pdx-cre mouse. KPC cells were cultured in RPMI media supplemented with 10% FBS, 2 mmol/L L-glutamine, 1% penicillin/streptomycin, 1% MEM non-essential amino acids, 1 mmol/L sodium pyruvate, and 0.1 mmol/L β-mercaptoethanol. All cells were cultured at 37°C in a humidified incubator with 5% CO_2_.

### Clonogenic Survival Assay

Actively growing monolayers of KPC and Panc-02 cancer cells were trypsinized and 300 cells per well were seeded in 6-well plates. 24 hours later, seeded cells were treated with 0, 1, 2, or 4 μM of FBL-03G concentrations per well. The cells were irradiated at 0, 2, or 4 Gy using 220 kVp energy, 13 mA, 24 h after the FBL-03G treatment. A small animal radiation research platform (SARRP) was used to deliver external beam radiation. The growing colonies (≥ 50 cells/colony) were fixed with 75% ethanol and stained with 1% crystal violet 9–12 days after treatment. Colonies were counted using ImageJ software and a percent survival was calculated following standard protocol.

### Mice and Generation of Syngeneic Pancreatic Cancer Models

Wild-type C57BL/6 strain male and female mice were acquired from Taconic Biosciences, Inc. at 8-weeks old. Animal experiments followed the guidelines and regulations set by the Dana-Farber Cancer Institute Institutional Animal Care and Use Committee (IACUC). Mice maintenance in Dana-Farber Cancer Institute animal facility was in accordance with the Institutional Animal Care and Use Committee approved guidelines. All treatments were given directly to one tumor either by direct intra-tumoral injection or by intra-tumoral implantation of loaded SRB for sustain release. For cohorts treated in conjunction with radiotherapy, a Small Animal Radiation Research platform (SARRP) was used for image-guided radiation therapy at 220 kVp and 13 mA. The study design included a randomization process of the mice followed by assortment into the following cohorts of: no treatment, RT dose of 6 Gy, FBL-03G with/without 6Gy, and SRB loaded with FBL- 03G and with/without 6Gy. All mice that received FBL-03G treatment in the first and second trials were treated with 100-μg of FBL-03G immunoadjuvant. The same amount of payload was used in SRBs as with other administration routes. SRBs were administered in the right tumors using a 17-Gauge clinical brachytherapy needle. Dimethyl sulfoxide (DMSO) was used as a solvent to dissolve FBL-03G powder. To investigate the potential of FBL-03G as an immunotherapy, different concentrations for FBL-03G in the amount of 100, 200, and 300 μg were considered. Same procedure of drug loading into SRBs were followed as the first and second trials. Tumor volumes were measured for both tumors on day of treatment and at least 1–2 times/week post-treatment. A survival study was also performed. Mice were euthanized when either tumor exceeded 20 mm in diameter collectively and/or when tumors were ulcerated or ruptured. A control cohort was created with no treatment of FBL-03G but an inoculation of DMSO.

In 3 independent animal studies, the mice were randomized and divided into some the following cohorts of: no treatment, 6 Gy, FBL-03G with/without 6Gy, and SRB loaded with/without FBL-03G and with/without 6Gy. Mice inoculated with FBL-03G treatment received either 100, 200, or 300 ug of FBL-03G. Payload of the same amount of FBL-03G was used in SRBs as with other administration routes.

### Tumor Volume Assessment

Directly after treatment, a digital Vernier caliper was used to measure the length and width of the dermal tumors. Tumor volume formula used: (length × width^2^)/2. Measurement imaginary longitude to the leg was chosen as length and the vertical was for width. The tumors were restrained between the skin surface layers. The tumor volume was plotted against time. Animal survival was performed for treatments following IACUC-approved protocol, which was predetermined based on published evidence justifying such a study design. Tumor attainment > 1 cm in diameter on both flanks or tumor burst were determined as excessive tumor burden and mouse was euthanized following the protocol.

### Statistical Analysis

The *in-vitro* experiments were conducted in triplicate, and data were presented as mean ± standard error or in the form quantified otherwise. Mice tumor volume were scrutinized using standard Student's two-tailed *t*-test. Mice survival were analyzed using GraphPad Prism 8.0. A *p*-value of ^*^P < 0.05, ^**^P < 0.01, ^***^P < 0.001, and ^****^P < 0.0001 were deemed as statistically significant difference.

## Results

**Figure 1:**
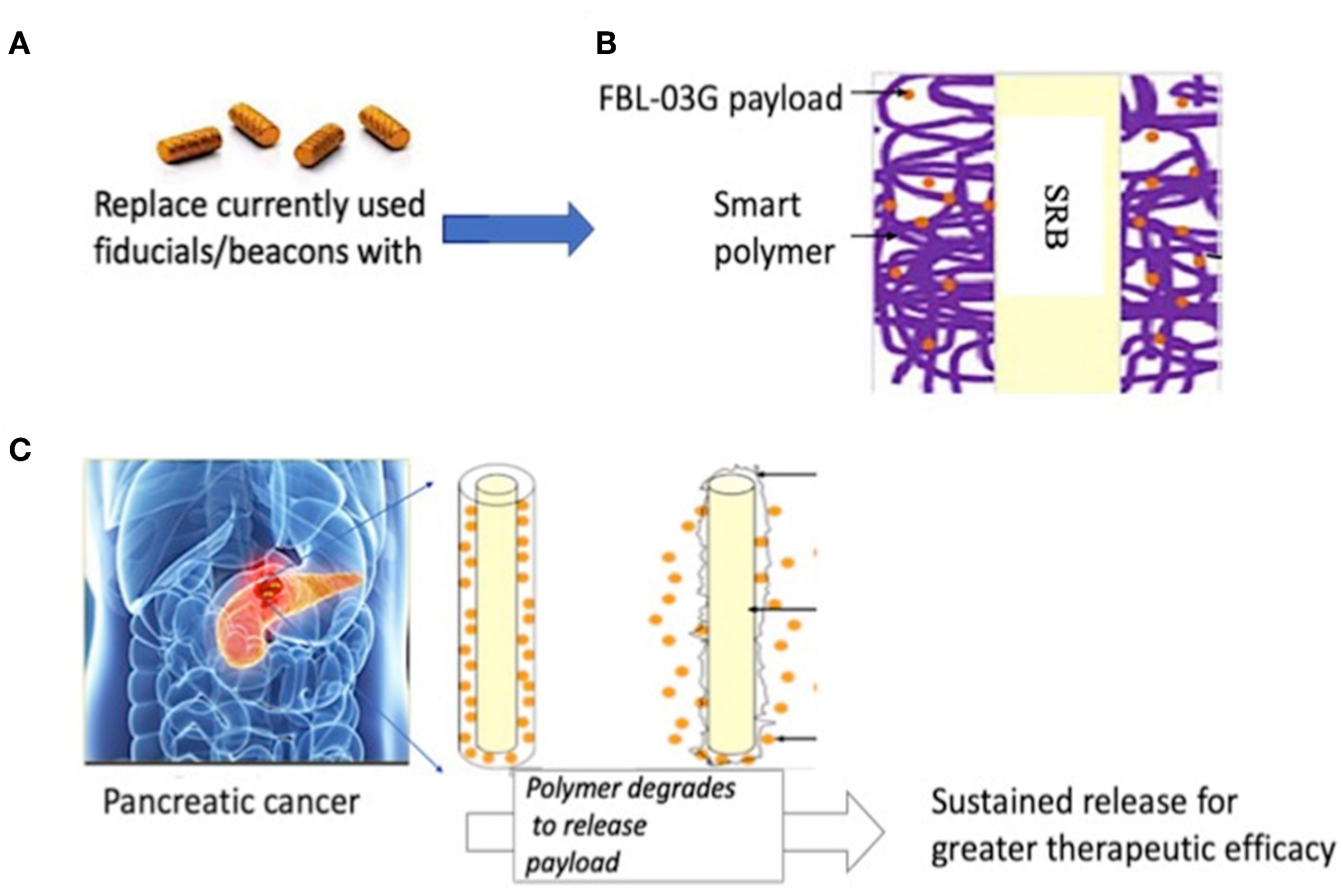
Illustration of pancreatic therapy approach using non-cannabinoid cannabis derivative, FBL-03G. **(A)** Currently used commercially-available inert radiotherapy biomaterials e.g., fiducial (CIVCO Medical)/beacons used during radiotherapy to ensure geometric accuracy **(B)** smart radiotherapy biomaterials (SRB) with FDA approved polymer component loaded with FBL-03G; **(C)** potential clinical translation pathway is envisioned where the SRBs could simply replace the inert biomaterials (in **A**). Such replacement would come at no additional inconvenience to cancer patients. Once in place the SRBs can be activated by tumor microenvironment to sustainably release FLB-03G as the polymer component degrades for greater effective tumor cell kill, potentially with or without radiotherapy.

**Figure 2:**
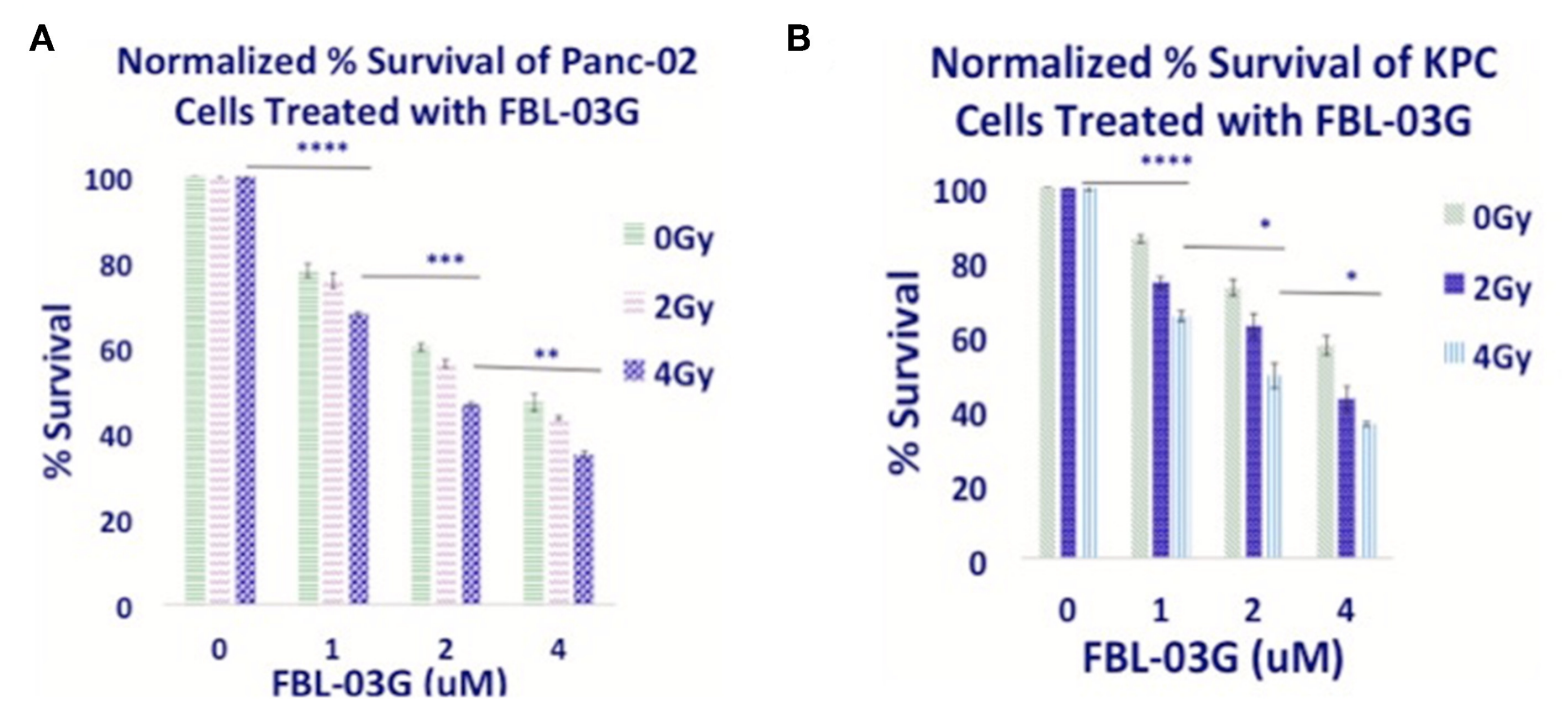
*In-vitro* anti-cancer effect of FBL-03G. FBL-03G drug from flavonoids shows anti-cell proliferation effects in combination with radiotherapy. Average results of normalized clonogenic assays are shown, respectively, for Panc-02 and KPC cells. **(A,B)** Results of synergistic outcomes when combining radiotherapy at 4Gy with different FBL-03G doses. Statistics is shown for cohorts treated at 4Gy at different doses of FBL-03G. Statistical Analyses using Student's *T*-Test for the Cell % survival at different concentrations of FBL-03G graphs (*n* = 3 independent trials), (^*^P < 0.05; ^**^P < 0.01; ^***^P < 0.001; ^****^P < 0.0001).

Figure 1 illustrates the therapy approach using FBL-03G loaded in smart radiotherapy biomaterials (SRBs) for sustained delivery to tumor cells. Before *in-vivo* studies with the FBL-03G, *in-vitro* studies were carried out with sustained exposure of cancer cells with FBL-03G. Clonogenic assay was performed to identify the anti-cancer effect of the FBL-03G drug with and without radiotherapy on 2 pancreatic cancer cell lines, KPC and Panc-02. Figure 2 highlights enhanced tumor cell death for the combination treatment of FBL-03G and radiation compared to individual treatments alone. The clonogenic survival results show that the use of 1 μM of FBL-03G has synergistic effect on pancreatic cells with exposure to 4 Gy of radiotherapy in terms of decreasing pancreatic cancer cell proliferation. These findings were observed for both Panc-02 and KPC pancreatic cancer cell lines. This demonstrates therapy potential for the FBL-03G. Moreover, the use of 4 μM of FBL-03G was apparently more effective in killing pancreatic cancer cells than 4 Gy of radiotherapy. This suggests that FBL-03G can induce apoptosis and inhibit cancer cell proliferation with optimized drug concentrations. The FBL-03G effect on cancer cells combined with DNA damage from radiotherapy could account for the observed synergistic outcomes.

**Figure 3:**
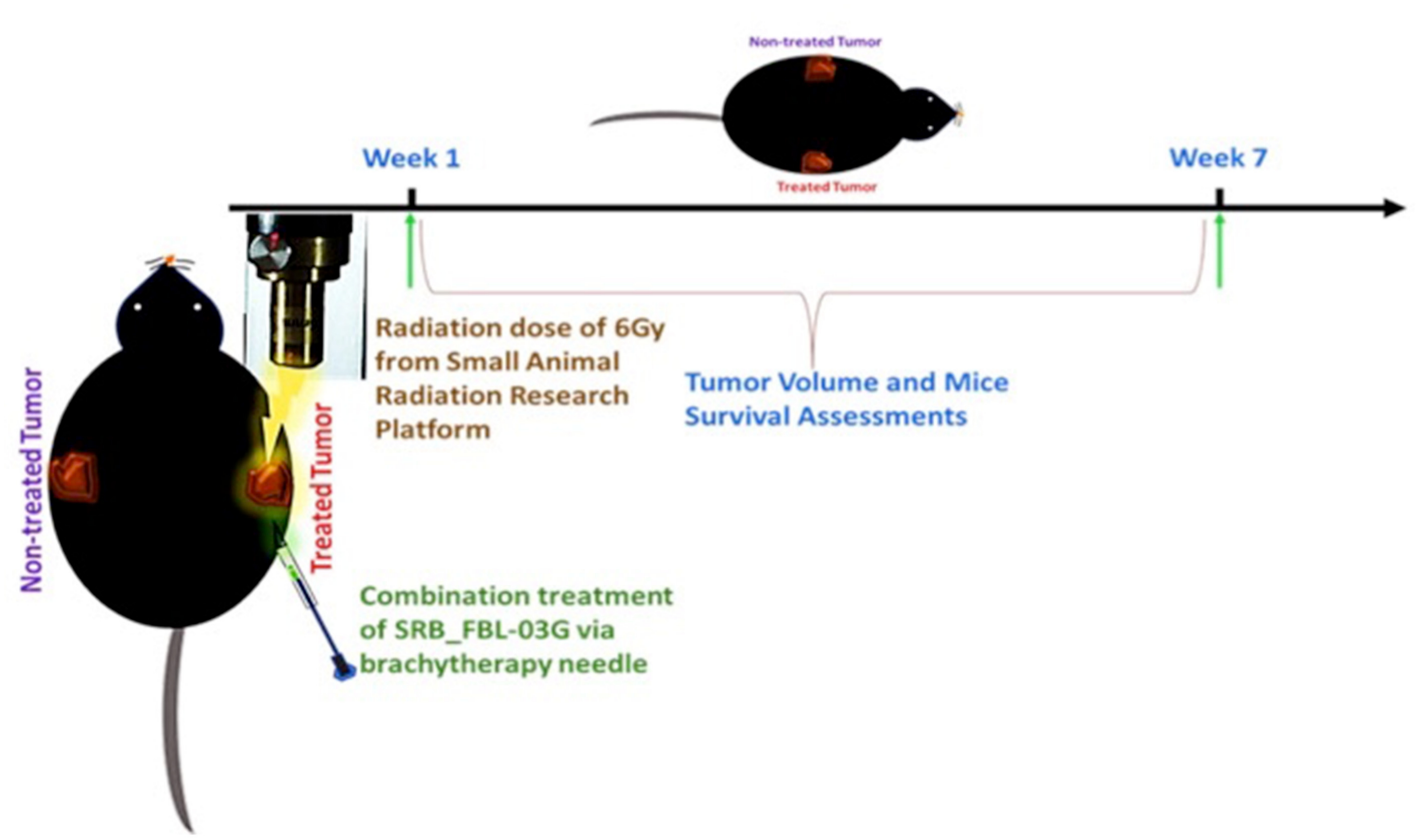
Schematic diagram showing the treatment design with radiotherapy and/or intra-tumor injection of FBL-03G/SRB_FBL-03G for subcutaneously inoculated pancreatic adenocarcinoma tumors. Week 1 represents the treatment week as depicted above after pancreatic cancer (KPC) tumor reached 3.5–4 mm in diameter. The treatment parameters included a single fraction of 6Gy dose of radiotherapy followed by either an inoculation of flavocure drug (FBL-03G) or an implant of smart radiotherapy biomaterials loaded with similar concentration of the drug, FBL-03G. Tumor volume and mice survival were assessed for up to 7-weeks post treatment.

**Figure 4:**
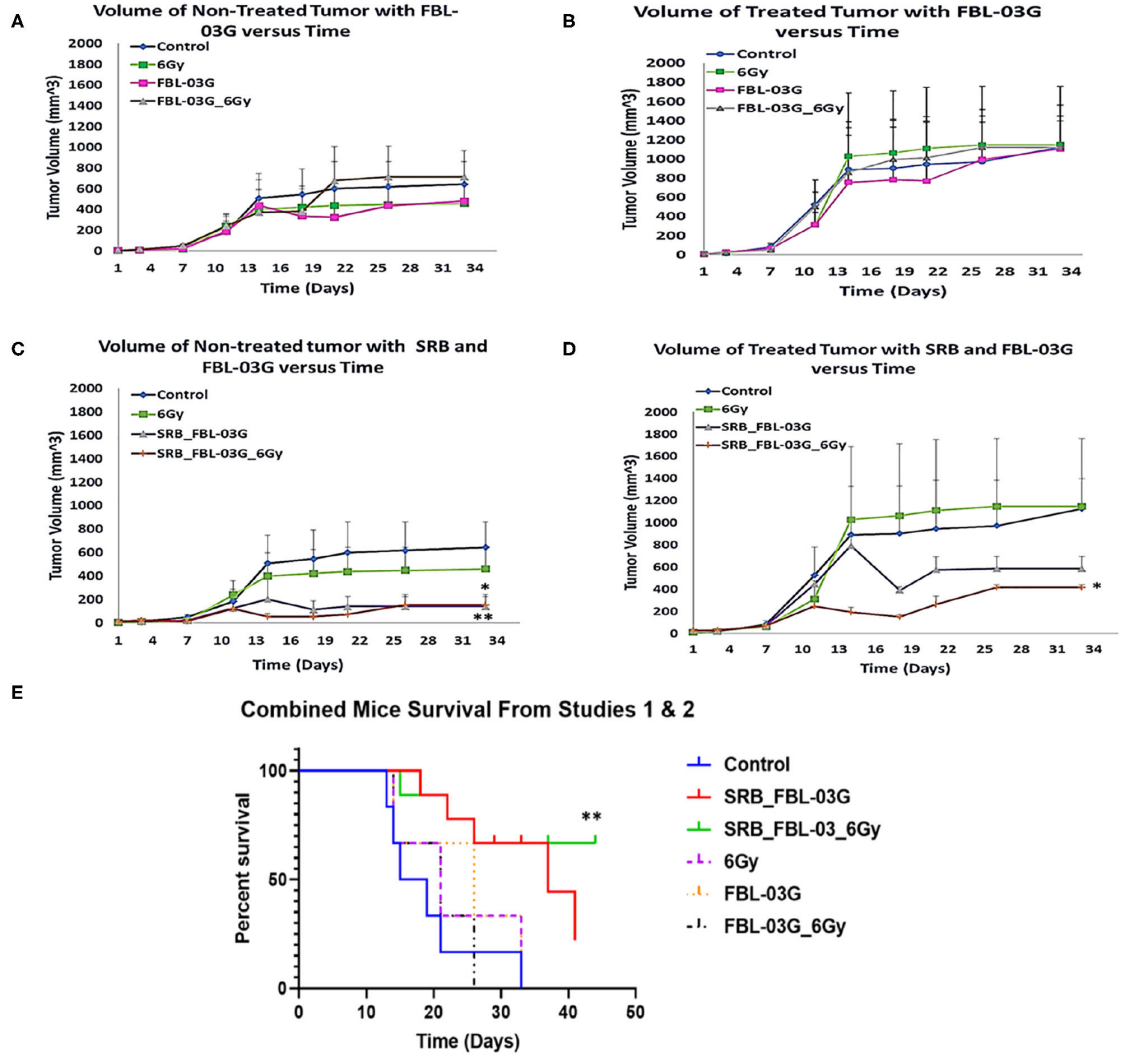
*In-vivo* treatment of C57BL/6 mice. Mice were inoculated with 50 μL of KPC cells in PBS suspension at concentrations of 5 × 10^4^ pancreatic cancer cells, on each left and right flank of mouse using a 22-Gauge syringe. When right side tumors reached palpable size, mice were randomized and treatments were administered. Mice were observed at least twice per week and tumor measurements were performed using precision calipers at least once per week. The abscopal effect was examined by monitoring the non-treated tumor. Smart radiotherapy biomaterials (SRB) loaded with FBL-03G (100 μg) significantly boosts the abscopal effect in pancreatic cancer slowing down tumor growth for both treated and untreated tumors. Two experiments were conducted simultaneously: Study 1 results are shown in graphs **(A–D)** and combined survival results for study 1 and study 2 results are displayed in **(E)**. **(A)** Volumes of non-treated tumors over time without SRB (*n* = 3 for each cohort). **(B)** Volumes of treated tumors over time (*n* = 3 for each cohort). **(C)** Volume of non-treated tumors over time with SRB and FBL-03G (*n* = 3 for control and 6Gy cohorts respectively; *n* = 4 for SRB loaded with FBL-03G with/without radiotherapy cohorts respectively). **(D)** Volume of treated tumors over time for cohorts treated with SRB and FBL-03G (*n* = 3 for control and 6 Gy cohorts respectively; *n* = 4 for SRB loaded with FBL-03G with/without radiotherapy cohorts respectively). **(E)** Survival results show significant increase in survival for cohorts treated with SRB loaded with FBL-03G (each *n* = 9) compared to control (*n* = 6), 6Gy/FBL-03G/FBL-03G_6Gy (each *n* = 3). For Statistical Analyses (*P < 0.05; **P < 0.01) Student's *T*-Test was used for comparing the volumes of tumors for each treatment group versus those of the control group with no additional corrections, and Log-rank (Mantel-Cox) was used for the survival graphs.

Using smart radiotherapy biomaterials for prolonged delivery of FBL-03G into tumors can also boost malignant cell death *in-vivo* with FBL-03G. The *in-vivo* study design is illustrated in Figure 3. The results are shown in Figure 4. Figures 4A–D shows a distinction between direct intra-tumor injection of FBL-03G vs. using the same concentration of FBL-03G with the smart radiotherapy biomaterial platform. Figures 4C,D shows reduction of tumor growth in animal cohorts treated with FBL-03G loaded in SRB compared to cohorts treated with direct administration of the same dose of FBL-03G shown in Figures 4A,B vs. control and irradiated cohorts. Remarkably, the results in Figure 4D revealed that nearby non-treated (abscopal) tumors, representing metastasis, were also significantly affected with slowed tumor growth. Repeated experiments showed significant increase in mice survival (Figures 4E,F) compared to control cohorts. The findings provide a basis for further studies to optimize different parameters for maximal outcomes via this approach.

**Figure 5:**
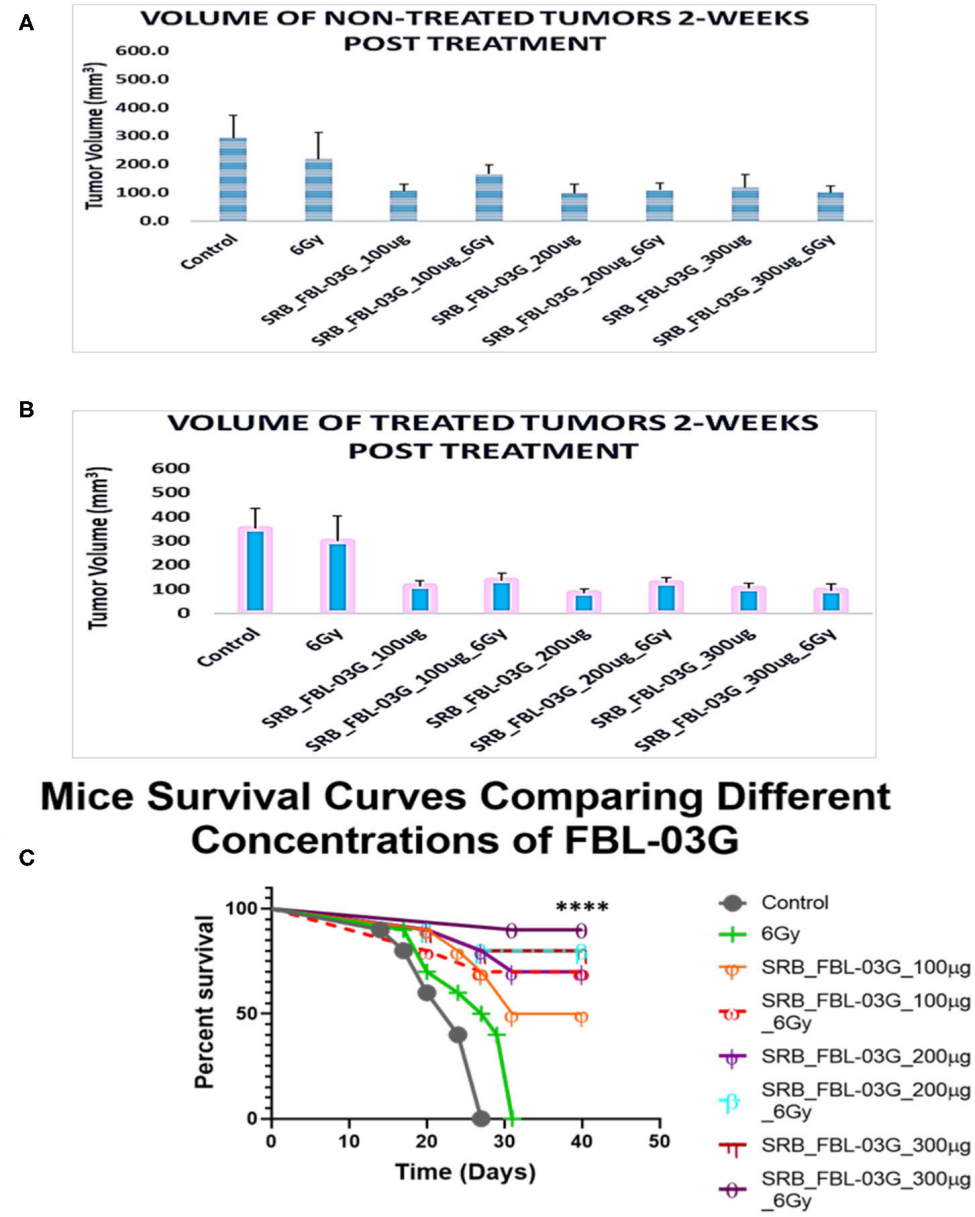
Investigating the optimal concentration of FBL-03G loaded in SRB to enhance the abscopal effect. Smart radiotherapy biomaterial (SRB) loaded, respectively, with FBL-03G (100, 200, or 300 μg). C57BL/6 mice were inoculated with pancreatic cancer cells (KPC) on both flanks. Tumor volume and survival (*n* = 10 for each cohort) were assessed. **(A)** Volumes of non-treated tumors 2-weeks post treatment (*n* = 10 for each cohort); **(B)** volumes of treated tumors 2-weeks post treatment (*n* = 10 for each cohort). This study investigated using different concentrations of FBL-03G with/without 6Gy to determine its potential effect on mice survival over time. **(C)** Represents a Log-rank (Mantel-Cox) survival graph (*n* = 10) (****p < 0.0001). **(C)** Survival results show no difference in survival for cohorts treated with different concentrations of SRB loaded with FBL-03G.

In another study we evaluated the effect of FBL-03G using 3 different concentrations (100, 200, and 300 ug) loaded in smart radiotherapy biomaterials with/without radiotherapy. The findings in Figure 5 show no significant difference in tumor volume between using smart radiotherapy biomaterials with FBL-03G alone vs. using SRBs with FBL-03G payloads in combination with radiotherapy. However, a significant difference between 6Gy and control cohorts vs. combination of FBL-03G in SRB treated groups is observed in Figures 5A,B, where tumor growth of both treated and non-treated tumors is inhibited compared to control and 6Gy cohorts. Overall, the data demonstrates significant therapeutic potential for using FBL-03G in the treatment of both local and metastatic disease, significantly increasing survival (Figure 5C).

## Discussion

From the results of this study, the key findings include, observation that a non-cannabinoid derivative of cannabis can enhance radiotherapy treatment outcomes *in-vitro* and *in-vivo* as highlighted in Figures 2, 4. Secondly, the sustained delivery of the cannabis derivative FBL-03G from smart radiotherapy biomaterials (SRBs) results in tumor growth inhibition of both locally treated and distant untreated tumors, with and without radiotherapy. The use of smart radiotherapy biomaterials (SRBs) (8, 23) was recently proposed as a novel approach to deliver cannabinoids, allowing for prolonged exposure of tumor cells to these cannabis derivatives, which is expected to be more effective (10). The FBL-03G payload used in this study is a flavonoid non-cannabinoid derivative of cannabis, and the potential to inhibit both local and metastatic tumor progression is remarkable, especially for pancreatic cancer, with a dismal 5-year survival rate of 8% (1).

While ongoing studies are in progress to address the specific mechanism for this immunotherapy potential of this cannabis derivative, the possibility of leveraging such a therapy approach to treat metastasis or increase survival is significant, given that most pancreatic cancer patients are diagnosed already with metastatic disease, with limited treatment options. The results highlight the potential of using non-cannabinoid/non-psychoactive derivatives of cannabis for such treatment. Further work to optimize therapeutic efficacy for such cannabis derivatives and evaluate toxicity could set the stage for clinical translation. An advantage of the SRB approach here is also that this could minimize any toxicity due to *in-situ* delivery and use of multifold less immunoadjuvant. Furthermore, the use of a single dose of RT as done in this project would minimize normal tissue toxicity.

Although Figure 4 shows no significant difference between using SRBs alone vs. SRBs with RT, SRBs could simply be used like fiducial markers (23) (Figure 1) offering a viable pathway to clinical translation at no additional inconvenience to patients. Another advantage of using SRBs for sustained *in-situ* delivery of payloads is the relative convenience in delivering the immunoadjuvants, compared to repeated injections. Using only one fraction of RT would also be more convenient for cancer patients who usually must come in repeatedly over many weeks to be treated with several fractions of radiotherapy. This should significantly reduce treatment time and costs. It would be a benefit in resource-poor-settings where access to RT services is limited, reducing cancer health disparities, with major impact in global health.

While the results indicate that sustained exposure of tumor cells to FBL-03G can boost both local and metastatic tumor cell kill, the mechanism of such action needs to be further investigated. One hypothesis is that, FBL-03G can serve as an immunotherapy agent, inhibiting growth of locally treated and untreated tumors, representing metastasis. Metastasis accounts for most of all cancer associated suffering and death, and questionably presents the most daunting challenge in cancer management. Henceforth, the observed significant increase in survival is promising, especially for pancreatic cancer which is often recalcitrant to treatments. Another hypothesis is that sustained delivery allows FBL-03G to reach the untreated tumor over a prolonged period as well. Either way, the FBL-03G results reveal a new potential non-cannabinoid cannabis derivative with major potential for consideration in further investigations in the treatment of pancreatic cancer, where new therapy options are urgently needed.

## Conclusion

In this study, a flavonoid derivative of cannabis demonstrates significant therapy potential in the treatment of pancreatic cancer, including radio-sensitizing and cancer metastasis treatment potential. The results justify further studies to optimize therapy outcomes toward clinical translation.

## Data Availability

All datasets generated for this study are included in the manuscript and/or the Supplementary Files.

## Ethics Statement

Animal experiments and protocol followed the guidelines and regulations set by the Dana-Farber Cancer Institute Institutional Animal Care and Use Committee (IACUC). Mice maintenance in Dana Farber Cancer Institute animal facility was in accordance with the Institutional Animal Care and Use Committee approved guidelines.

## Author Contributions

MM provided intellectual contributions to the design of the mice study, generated all the results in this study, designed the smart radiotherapy biomaterial (SRB) to implant in mice, and wrote most of the manuscript. SY-K reviewed the manuscript. UI, KD, and NB helped in tumor measurements for mice and reviewed the manuscript. NT and HL provided the FBL-03G drug, contributed to study design, made input in manuscript including reviewing the manuscript. WN is the principal investigator who designed the study and wrote a portion of the manuscript.

Funding support for this work is acknowledged from the USA National Institutes of Health (NIH), grant number R21CA205094 and Flavocure Biotech Inc.

### Conflict of Interest Statement

NT and HL work for Flavocure Biotech Inc., a for-profit company. The remaining authors declare that the research was conducted in the absence of any commercial or financial relationships that could be construed as a potential conflict of interest.

## Supplementary Material

Figure S1 Structures of Cannabis flavonoids and the unnatural molecule FBL-03G. Through a bioactivity guided isolation approach, Cannflavin B (**10**) was isolated from a flavonoid rich fraction of Cannabis using flash chromatography along with 10 other compounds and characterized by NMR and MS spectroscopic methods. The spectroscopic data were similar to the data of the following 11 compounds previously isolated and characterized from the Cannabis plant; apigenin (**1**), Chrysoeriol (**2**), kaempferol (**3**), luteolin (**4**), quercetin (**5**), vitexin (**6**), isovitexin (**7**), orientin (**8**) and prenylated flavonoids including Cannflavin A (**9**), Cannflavin B (**10**), and Cannflavin C (**11**).
