# Corrigendum: Flavonoid Derivative of *Cannabis* Demonstrates Therapeutic Potential in Preclinical Models of Metastatic Pancreatic Cancer

**DOI:** 10.3389/fonc.2020.01434

**Published:** 2020-08-21

**Authors:** Michele Moreau, Udoka Ibeh, Kaylie Decosmo, Noella Bih, Sayeda Yasmin-Karim, Ngeh Toyang, Henry Lowe, Wilfred Ngwa

**Affiliations:** ^1^Brigham and Women's Hospital, Dana-Farber Cancer Institute, Harvard Medical School, Boston, MA, United States; ^2^Department of Physics, University of Massachusetts Lowell, Lowell, MA, United States; ^3^Department of Biology, University of Massachusetts Boston, Boston, MA, United States; ^4^Department of CaNCURE Program, Northeastern University, Boston, MA, United States; ^5^Flavocure Biotech Inc., Baltimore, MD, United States

**Keywords:** pancreatic cancer, flavonoids, cannabis, metastasis, radiotherapy, smart biomaterials

In the original article, there was a mistake in Figures 4E, 4F, and 5C as published. This was due to errors during use of analysis software. The survival data in Figures 4E,F has been combined into one Figure 4E. The figure legend of Figure 4 has been updated to reflect the correction made in the figure. The corrected Figures 4 and 5 appear below.

**Figure 4:**
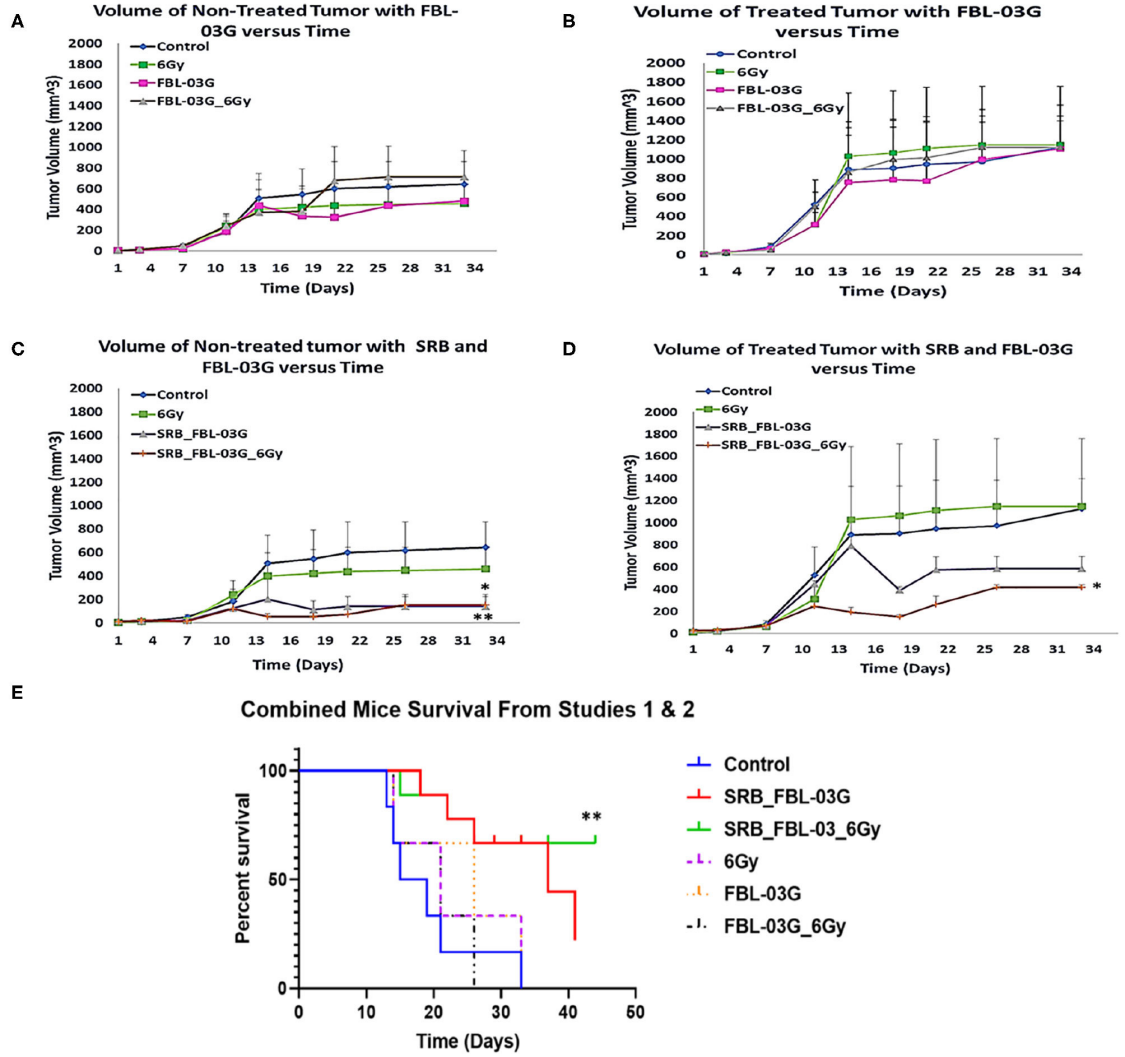
*In-vivo* treatment of C57BL/6 mice. Mice were inoculated with 50 μL of KPC cells in PBS suspension at concentrations of 5 × 10^4^ pancreatic cancer cells, on each left and right flank of mouse using a 22-Gauge syringe. When right side tumors reached palpable size, mice were randomized and treatments were administered. Mice were observed at least twice per week and tumor measurements were performed using precision calipers at least once per week. The abscopal effect was examined by monitoring the non-treated tumor. Smart radiotherapy biomaterials (SRB) loaded with FBL-03G (100 μg) significantly boosts the abscopal effect in pancreatic cancer slowing down tumor growth for both treated and untreated tumors. Two experiments were conducted simultaneously: Study 1 results are shown in graphs **(A–D)** and combined survival results for study 1 and study 2 results are displayed in **(E)**. **(A)** Volumes of non-treated tumors over time without SRB (*n* = 3 for each cohort). **(B)** Volumes of treated tumors over time (*n* = 3 for each cohort). **(C)** Volume of non-treated tumors over time with SRB and FBL-03G (*n* = 3 for control and 6Gy cohorts respectively; *n* = 4 for SRB loaded with FBL-03G with/without radiotherapy cohorts respectively). **(D)** Volume of treated tumors over time for cohorts treated with SRB and FBL-03G (*n* = 3 for control and 6 Gy cohorts respectively; *n* = 4 for SRB loaded with FBL-03G with/without radiotherapy cohorts respectively). **(E)** Survival results show significant increase in survival for cohorts treated with SRB loaded with FBL-03G (each *n* = 9) compared to control (*n* = 6), 6Gy/FBL-03G/FBL-03G_6Gy (each *n* = 3). For Statistical Analyses (*P < 0.05; **P < 0.01) Student's *T*-Test was used for comparing the volumes of tumors for each treatment group versus those of the control group with no additional corrections, and Log-rank (Mantel-Cox) was used for the survival graphs.

**Figure 5:**
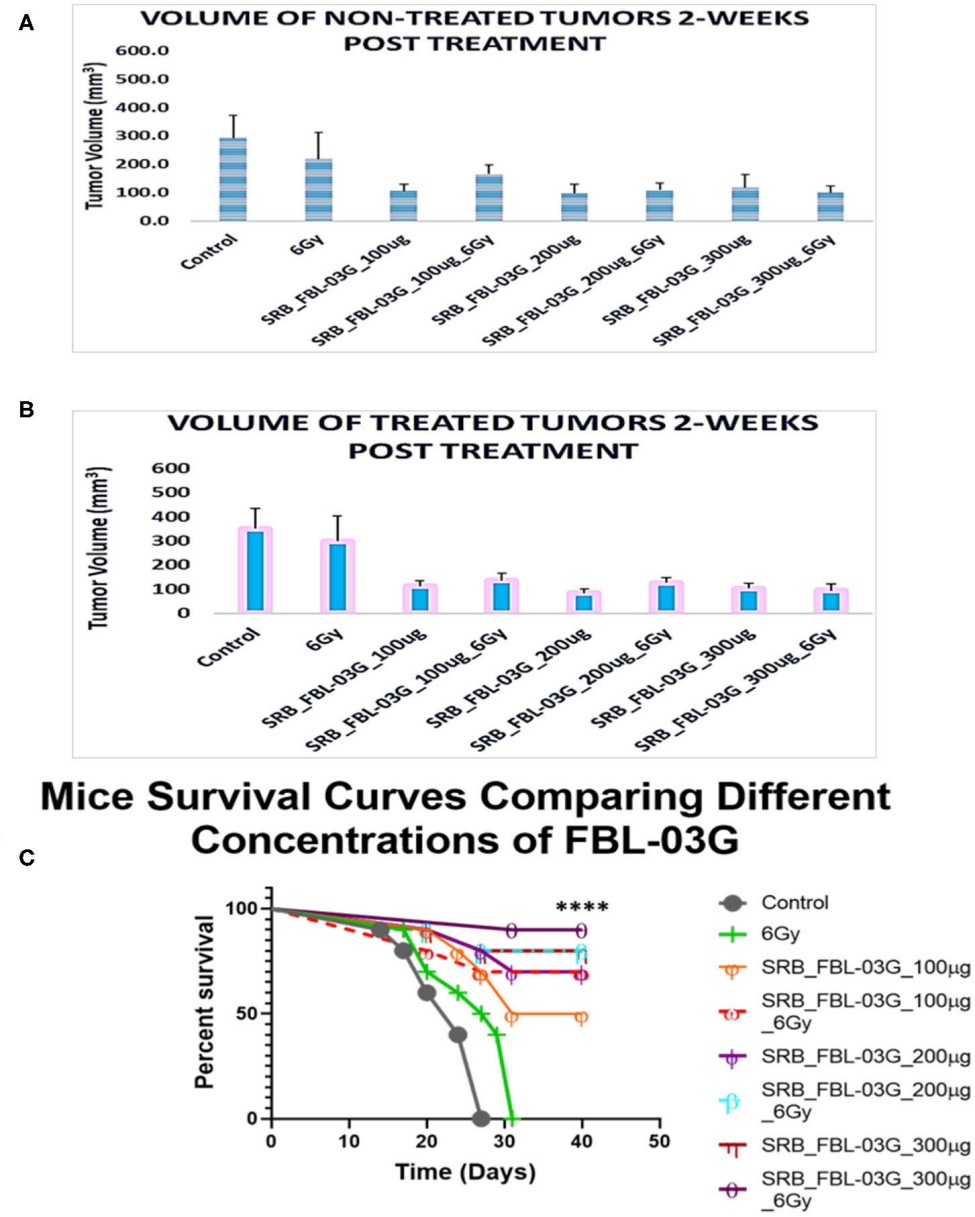
Investigating the optimal concentration of FBL-03G loaded in SRB to enhance the abscopal effect. Smart radiotherapy biomaterial (SRB) loaded, respectively, with FBL-03G (100, 200, or 300 μg). C57BL/6 mice were inoculated with pancreatic cancer cells (KPC) on both flanks. Tumor volume and survival (*n* = 10 for each cohort) were assessed. **(A)** Volumes of non-treated tumors 2-weeks post treatment (*n* = 10 for each cohort); **(B)** volumes of treated tumors 2-weeks post treatment (*n* = 10 for each cohort). This study investigated using different concentrations of FBL-03G with/without 6Gy to determine its potential effect on mice survival over time. **(C)** Represents a Log-rank (Mantel-Cox) survival graph (*n* = 10) (****p < 0.0001). **(C)** Survival results show no difference in survival for cohorts treated with different concentrations of SRB loaded with FBL-03G.

The data for the tumor volume and survival results has also now been published as Supplementary Material.

## Supplementary Material

The authors apologize for this error and state that these do not change the scientific conclusions of the article in any way. The original article has been updated.

