# Supplementary figures and images for: Flavonoid Derivative of Cannabis Demonstrates Therapeutic Potential in Preclinical Models of Metastatic Pancreatic Cancer
Source: Front Oncol. 2019 Jul 23;9:660. doi: 10.3389/fonc.2019.00660 (PMC6663976; doi:10.3389/fonc.2019.00660)

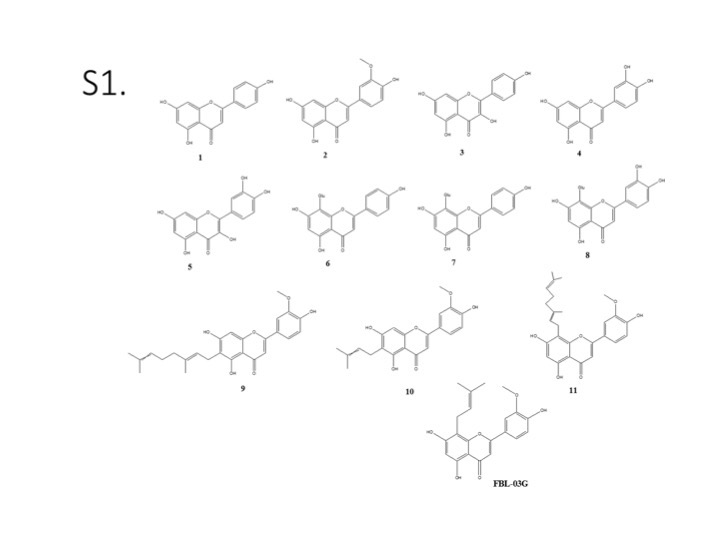

Supplement: Figure S1 — Structures of Cannabis flavonoids and the unnatural molecule FBL-03G. Through a bioactivity guided isolation approach, Cannflavin B (10) was isolated from a flavonoid rich fraction of Cannabis using flash chromatography along with 10 other compounds and characterized by NMR and MS spectroscopic methods. The spectroscopic data were similar to the data of the following 11 compounds previously isolated and characterized from the Cannabis plant; apigenin (1), Chrysoeriol (2), kaempferol (3), luteolin (4), quercetin (5), vitexin (6), isovitexin (7), orientin (8) and prenylated flavonoids including Cannflavin A (9), Cannflavin B (10), and Cannflavin C (11). [file Image_1.JPEG]

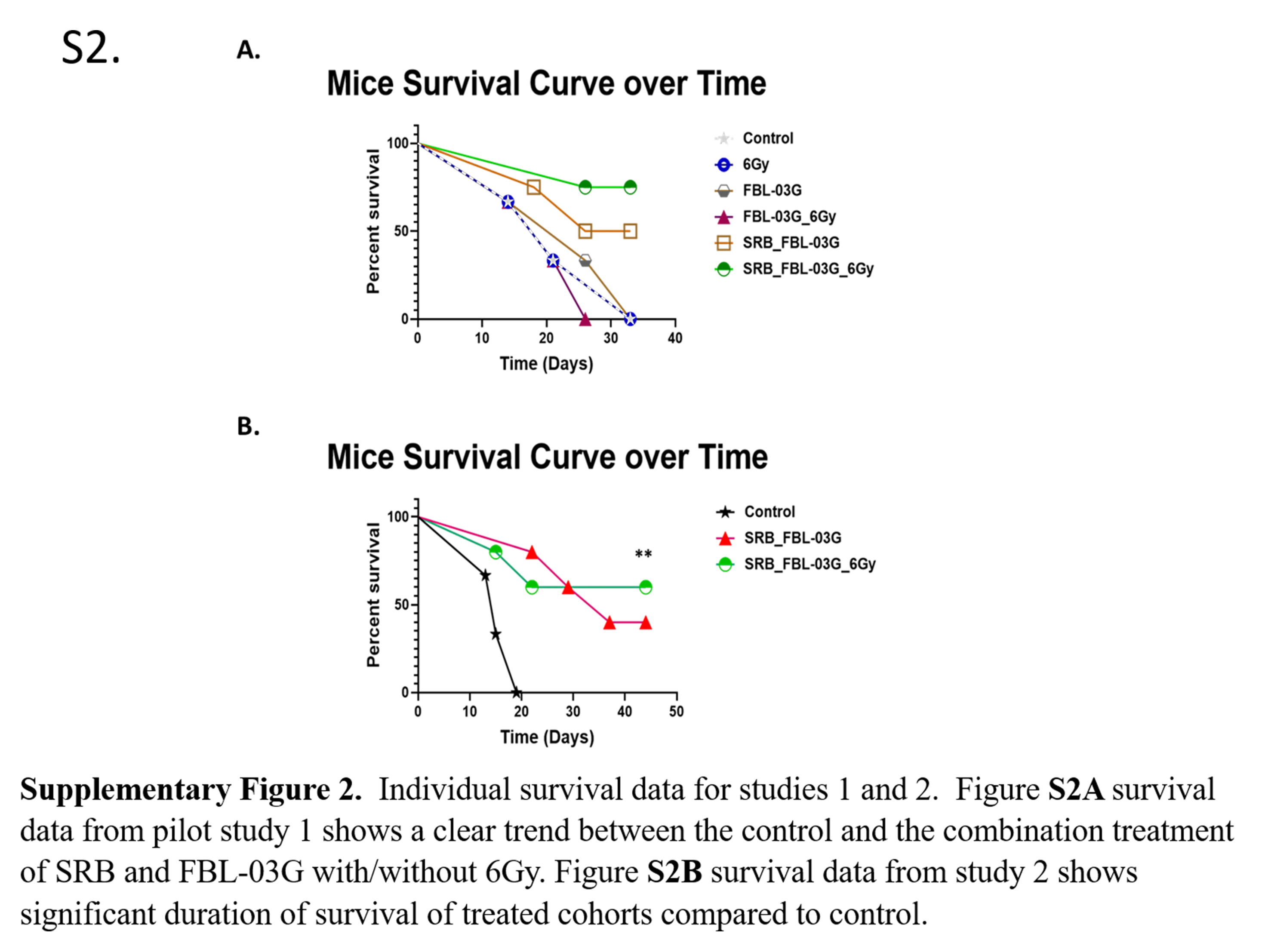

Supplement: Supplementary file 2 [file Image_2.png]
