# Supplementary material for: Flavonoid Derivative of Cannabis Demonstrates Therapeutic Potential in Preclinical Models of Metastatic Pancreatic Cancer
Source: Front Oncol. 2019 Jul 23;9:660. doi: 10.3389/fonc.2019.00660 (PMC6663976; doi:10.3389/fonc.2019.00660)
Supplement: Supplementary file 3 [file Data_Sheet_1.pdf]

Figure S2A Raw Survival Data

Days Post Treatment	Control Group	Dead
1	3	0
3	3	0
7	3	0
11	3	0
14	3	-1
18	2	0
21	2	-1
26	1	0
29	1	0
33	1	-1
**Days Post Treatment**	**6Gy Group**	**Dead**
1	3	0
3	3	0
7	3	0
11	3	0
14	3	-1
18	2	0
21	2	-1
26	1	0
29	1	0
33	1	-1

NOTE:

- **Initial # of mice for each group is indicated on day 1.**
- **The negative values are indicative of when mice reach the endpoint of the study.**

Days Post Treatment	FBL-03G Group	Dead
1	3	0
3	3	0
7	3	0
11	3	0
14	3	-1
18	2	0
21	2	0
26	2	-1
29	1	0
33	1	-1
Days Post Treatment	FBL-03G_6Gy Group	Dead
1	3	0
3	3	0
7	3	0
11	3	0
14	3	-1
18	2	0
21	2	-1
26	1	-1
29	0	0
33	0	0

Days Post Treatment	SRB_FBL-03G Group	Dead
1	4	0
3	4	0
7	4	0
11	4	0
14	4	0
18	4	-1
21	3	0
26	3	-1
29	2	0
33	2	0
**Days Post Treatment**	**SRB_FBL-03G_6Gy Group**	**Dead**
1	4	0
3	4	0
7	4	0
11	4	0
14	4	0
18	4	0
21	4	0
26	4	-1
29	3	0
33	3	0

Figure S2B Raw Survival Data

Days Post Treatment	Control Group	Dead
**0**	3	0
**4**	3	0
**8**	3	0
**11**	3	0
**13**	3	0
**15**	3	-1
**19**	2	-1
**22**	1	-1
**25**	0	0
**29**	0	0
**33**	0	0
**37**	0	0
**41**	0	0
**44**	0	0
Days Post Treatment	SRB_FBL-03G Group	Dead
**0**	5	0
**4**	5	0
**8**	5	0
**11**	5	0
**13**	5	0
**15**	5	0
**19**	5	0
**22**	5	-1
**25**	4	0
**29**	4	-1
**33**	3	0
**37**	3	-1
**41**	2	0
**44**	2	0

NOTE:

- **Initial # of mice for each group is indicated on day 1.**
- **The negative values are indicative of when mice reach the endpoint of the study.**

**Days Post Treatment**	**SRB_FBL-03G_6Gy Group**	**Dead**
**0**	5	0
**4**	5	0
**8**	5	0
**11**	5	0
**13**	5	0
**15**	5	-1
**19**	4	0
**22**	4	-1
**25**	3	0
**29**	3	0
**33**	3	0
**37**	3	0
**41**	3	0
**44**	3	0
