# Supplementary material for: Flavonoid Derivative of Cannabis Demonstrates Therapeutic Potential in Preclinical Models of Metastatic Pancreatic Cancer
Source: Front Oncol. 2019 Jul 23;9:660. doi: 10.3389/fonc.2019.00660 (PMC6663976; doi:10.3389/fonc.2019.00660)
Supplement: Supplementary file 4 [file Data_Sheet_2.pdf]

Figures 4(A – D)
Average Tumor Volume(mm^3) Data

Treated Side

Cohorts	1	3	7	11	14	18	21	26	33
Control	8.669666667	16.16667	82.43961	525.2305	890.3089	902.464	945.1563	972.8423	1126.66
6Gy	10.103	17.70901	60.76485	311.6789	1027.932	1063.267	1110.526	1146.726	1148.116
FBL-03G	2.55	28.29716	63.21283	314.4973	755.3028	783.9444	772.1895	997.1033	1105.694
FBL-03G_6Gy	2.8125	24.137	47.80936	502.2751	859.6025	995.7102	1012.568	1122.159	
SRB_FBL-03G	27.15825	21.98497	69.13952	444.1768	793.1101	389.841	575.3216	584.4995	584.4995
SRB_FBL-03G_6Gy	22.137875	30.21365	70.7246	246.2044	191.8133	149.0191	261.575	417.1615	417.1615

Treated Side St. Error

Cohorts	1	3	7	11	14	18	21	26	33
Control	4.342927597	12.11175	31.62283	255.7837	437.476	430.1925	436.8106	410.4789	269.1814
6Gy	7.240994516	9.115705	19.64715	128.4032	659.3896	649.3557	639.9304	611.7698	610.7049
FBL-03G	0.099215674	7.791388	26.30385	209.6999	633.2858	618.8325	624.7533	518.5885	458.143
FBL-03G_6Gy	0.171846589	11.30518	23.21283	152.9867	387.4897	420.7045	428.2235	323.1988	
SRB_FBL-03G	7.836936143	4.011974	3.358922	32.43816	246.0869	32.48316	116.4248	110.1829	110.1829
SRB_FBL-03G_6Gy	6.228790755	8.701479	17.05325	43.17106	46.52029	23.57254	75.1455	23.28574	23.28574

Non-treated Side

Cohorts	1	3	7	11	14	18	21	26	33
Control	4.104166667	8.041667	47.11268	182.0675	507.1539	545.0583	599.8015	617.315	643.5688
6Gy	3.979166667	9.291667	17.34725	239.0654	399.3501	420.434	436.9513	446.1463	458.0713
FBL-03G	1.816666667	7.721141	24.33813	188.6834	437.5349	334.458	324.392	436.1148	481.6769
FBL-03G_6Gy	2.154166667	14.56831	49.31241	240.0207	372.2037	380.9455	678.6651	712.5115	
SRB_FBL-03G	13.15625	13.46875	19.02523	123.8093	202.7011	110.9271	138.9528	140.0778	140.0778
SRB_FBL-03G_6Gy	6.578125	19.03125	13.76768	120.4163	53.39203	52.47315	74.48345	152.8851	152.8851

Non-Treated Side St. Error

Cohorts	1	3	7	11	14	18	21	26	33
Control	2.23635135	6.160329	23.30648	103.3132	240.4588	245.9585	260.1969	242.7258	216.5483
6Gy	3.479166667	7.604167	7.284372	119.2877	198.0461	203.2161	207.5416	198.3738	186.488
FBL-03G	0.661529688	3.673035	12.3558	146.3292	247.1047	292.2052	297.4124	246.6886	218.8186
FBL-03G_6Gy	0.827720682	7.088509	14.64796	114.1869	213.7584	217.4886	326.6523	295.1142	
SRB_FBL-03G	12.49013934	12.39228	10.32514	81.91612	168.144	76.90608	83.29766	82.67966	82.67966
SRB_FBL-03G_6Gy	3.57867081	11.95438	9.898282	78.67515	24.96838	28.7021	42.32354	88.95545	88.95545

Figures 4(A – D)
Individual Tumor Volume (mm³) Data

Treated Side

Cohorts	Mouse#	1	3	7	11	14	18	21	26	33
Control	1	0.5	0.5	25.92	75	84.5325	109.76	111.6292	194.6872	656.1415
	2	10.2	8	86.11973	960.6915	997.9024	1009.14	1135.348	1135.348	1135.348
	3	15.309	40	135.2791	540	1588.492	1588.492	1588.492	1588.492	1588.492
6Gy	1	1.5895	0.5	24.5	68.75	81.12	93.7125	100.602	209.2015	213.3734
	2	24.5055	31.52655	91.99982	361.0233	706.3255	799.7382	934.626	934.626	934.626
	3	4.214	21.10048	65.79473	505.2633	2296.35	2296.35	2296.35	2296.35	2296.35
FBL-03G	1	2.3625	26.568	12.19701	100.316	99.32008	160.927	160.8988	624.8806	624.8806
	2	2.5875	42.57347	77.59792	109.3109	2021.6	2021.6	2021.6	2021.6	2021.6
	3	2.7	15.75	99.84358	733.865	144.9884	169.3061	134.0698	344.8294	670.6022
FBL-03G_6Gy	1	2.925	25.30061	41.81463	546.1635	646.9924	1184.656	1243.537	1243.537	1243.537
	2	2.475	4	10.9375	218.0903	320.5065	191.1658	182.86	511.6306	511.6306
	3	3.0375	43.1104	90.67594	742.5717	1611.309	1611.309	1611.309	1611.309	1611.309
SRB_FBL-03G	1	50.625	16.1472	68.64614	505.2261	1448.888	468.5209	468.5209	468.5209	468.5209
	2	20.6615	25.52435	62.01058	355.2521	858.2191	310.5851	629.9622	629.9622	629.9622
	3	18.424	14.65174	78.20773	475.4878	305.0278	380.3907	330.3809	368.0923	364.0923
	4	18.9225	31.6166	67.69363	440.7412	560.306	399.8672	872.4224	871.4224	875.4224
SRB_FBL-03G_6Gy	1	40.3065	50.625	113.1338	174.24	204.0781	81.82191	112.4118	400.7463	390.7463
	2	20.181	21.24209	51.26697	248.0479	80.67345	168.93	323.7899	362.2841	367.2841
	3	13.689	11.34	36.3443	367.0673	175.7564	190.5238	442.6388	434.3092	439.3092
	4	14.375	37.6475	82.1533	195.4622	306.7452	154.8009	167.4596	471.3064	471.3064

Non-Treated Side

Cohorts	Mouse#	1	3	7	11	14	18	21	26	33
Control	1	0.5	0.5	0.5	167.3492	655.5168	750.805	889.6772	889.6772	889.6772
	2	8.2	20.25	70.27769	10.9375	36.8	55.225	80.58266	133.1231	211.8846
	3	3.6125	3.375	70.56035	367.9159	829.1448	829.1448	829.1448	829.1448	829.1448
6Gy	1	0.5	1.6875	5	5	13.851	18	23.04	50.625	86.40007
	2	10.9375	24.5	30.21754	396.082	513.2987	572.4014	616.9134	616.9134	616.9134
	3	0.5	1.6875	16.8242	316.1143	670.9005	670.9005	670.9005	670.9005	670.9005
FBL-03G	1	2.3625	7.5	20.6176	178.03	319.144	175.8328	149.5147	326.8782	326.8782
	2	2.5875	6.163422	30.8968	309.375	618.6612	618.6612	618.6612	618.6612	618.6612
	3	0.5	9.5	21.5	78.64508	374.7995	208.88	205	362.8049	499.4912
FBL-03G_6Gy	1	2.925	23.12	67.04053	232.2854	241.3103	304.1981	1180.428	1180.428	1180.428
	2	0.5	0.5	20.25	46.2242	85.19022	48.52789	65.45689	166.9961	166.9961
	3	3.0375	20.08492	60.6467	441.5524	790.1106	790.1106	790.1106	790.1106	790.1106
SRB_FBL-03G	1	15.625	12.625	25.91343	358.3582	656.8417	336.9818	336.9818	336.9818	336.9818
	2	13	16.75	4	52.7575	50.625	28.88	150	205.6418	205.6418
	3	16	15	24.5	54.74665	78.83769	56.15928	50.5	11	10.6875
	4	8	9.5	21.6875	29.375	24.5	21.6875	18.3293	6.6878	7
SRB_FBL-03G_6Gy	1	5.9375	14.5	21.39092	42.6028	26.56731	45.91661	124.9742	267.3016	267.3016
	2	5.5	10.5	11	42.25	32.688	20.5	25.5	50	60
	3	7.5	25.5	10	62.25	52.9	22.935	40	54.1875	34.2386
	4	7.375	25.625	12.67981	334.5624	101.4128	120.541	107.4596	240.0511	250
