# Supplementary material for: Flavonoid Derivative of Cannabis Demonstrates Therapeutic Potential in Preclinical Models of Metastatic Pancreatic Cancer
Source: Front Oncol. 2019 Jul 23;9:660. doi: 10.3389/fonc.2019.00660 (PMC6663976; doi:10.3389/fonc.2019.00660)
Supplement: Supplementary file 5 [file Data_Sheet_3.pdf]

Individual Tumor volume for Figures 5(A-B)

	Tumor Volume (mm^3) 2-weeks Post Treatment		
		Abscopal Side	Treated Side
Cohorts	Mouse#	Individual Mouse Tumor Volume (mm^3)	
Control	1	901.1	866.2440465
	2	46.8	347.7152
	3	138.4	48.607488
	4	352.3	160.569154
	5	218.6	478.693028
	6	387.6	604.054575
	7	34.0	364.5
	8	94.6	493.802162
	9	345.4	46.8329125
	10	400.1	93.10935
6Gy	1	255.52555	358.0438875
	2	58.98629	371.793672
	3	346.625892	258.8142375
	4	35.8998255	63.335124
	5	15.5407	106.1705
	6	1021.680138	1176.264
	7	97.330496	81.817632
	8	38.241952	238.731342
	9	204.8049875	78.976337
	10	98.2064375	249.630921
SRB_100ug_FBL-03G	1	164.090144	76.43008
	2	72.60125	122.248976
	3	73.657188	247.2656
	4	178.8166925	36.341173
	5	59.83875	7.999186
	6	123.274679	176.8454025
	7	17.4746005	190.529302
	8	245.271699	80.842925
	9	13.454217	134.648568
	10	110.2304	43.814613
SRB_100ug_FBL-03G_6Gy	1	104.3240625	124.9318115
	2	53.87605	161.395
	3	130.72995	135.56976
	4	15.2662685	37.74186
	5	215.545806	147.459816
	6	181.863	90.943113
	7	368.44321	215.9209

	8	200.2138625	38.8044475
	9	206.560152	358.142796
	10	185.42986	50.68215
SRB_200ug_FBL-03G	1	83.77668	66.706983
	2	25.899208	49.683763
	3	119.159033	60.397625
	4	105.498048	196.187328
	5	113.433138	162.2168
	6	35.41564	41.150568
	7	361.3550215	95.9251005
	8	2.25	57.589056
	9	71.317132	103.8220525
	10	49.545144	5.84
SRB_200ug_FBL-03G_6Gy	1	8.199808	75.192325
	2	186.306606	117.328757
	3	104.152832	131.063387
	4	81.745204	38.364354
	5	88.6531785	186.7935605
	6	224.0711275	52.352522
	7	214.061688	98.555682
	8	38.2743	246.159816
	9	124.030332	211.7416055
	10	26.061312	101.3832135
SRB_300ug_FBL-03G	1	436.930281	114.989223
	2	15.06848	62.165475
	3	9.7862625	37.4918355
	4	9.396424	131.87133
	5	275.533596	178.9607625
	6	9.95982	121.0117635
	7	148.423248	47.18592
	8	151.1762455	233.0195435
	9	106.768656	82.001464
	10	9.766533	21.1725735
SRB_300ug_FBL-03G_6Gy	1	55.442925	21.1631875
	2	8.7362	172.4437755
	3	223.99769	37.5194985
	4	59.5767705	103.035694
	5	136.933984	169.8493995
	6	136.244556	277.595094
	7	217.728	23.4887965
	8	27.0817855	37.303

	9	8.283212	64.13904
	10	112.066308	25.3164

Average Tumor volume for Figures 5(A-B)

Average Tumor Volume (mm^3) 2-Weeks Post Treatment		
	Treated Side	Non-treated Side
Control	350	292
6Gy	298	217
SRB_FBL-03G_100ug	112	106
SRB_FBL-03G_100ug_6Gy	136	166
SRB_FBL-03G_200ug	84	97
SRB_FBL-03G_200ug_6Gy	126	110
SRB_FBL-03G_300ug	103	117
SRB_FBL-03G_300ug_6Gy	93	99
