# Supplementary material for: Flavonoid Derivative of Cannabis Demonstrates Therapeutic Potential in Preclinical Models of Metastatic Pancreatic Cancer
Source: Front Oncol. 2019 Jul 23;9:660. doi: 10.3389/fonc.2019.00660 (PMC6663976; doi:10.3389/fonc.2019.00660)
Supplement: Supplementary file 6 [file Data_Sheet_4.pdf]

Raw survival data for Figure 4E

Time (Days)	Control	SRB_FBL-03G	SRB_FBL-03_6Gy	6Gy	FBL-03G	FBL-03G_6Gy
14	-1					
21	-1					
33	-1					
13	-1					
15	-1					
19	-1					
18		-1				
26		-1				
33		0				
26		0				
22		-1				
29		0				
37		-1				
41		0				
41		-1				
26			-1			
33			0			
33			0			
33			0			
15			-1			
22			-1			
33			0			
37			0			
44			0			
14				-1		
21				-1		
33				-1		
14					-1	
26					-1	
33					-1	
14						-1
21						-1
26						-1

Table 1. The raw mice survival data shown above corresponds to Figure 4E.
