# Supplementary material for: Flavonoid Derivative of Cannabis Demonstrates Therapeutic Potential in Preclinical Models of Metastatic Pancreatic Cancer
Source: Front Oncol. 2019 Jul 23;9:660. doi: 10.3389/fonc.2019.00660 (PMC6663976; doi:10.3389/fonc.2019.00660)
Supplement: Supplementary file 7 [file Data_Sheet_5.pdf]

Raw survival data for Figure 5C

Time (Days)	Control	6Gy	SRB_FBL-03G_100µg	SRB_FBL-03G_100µg_6Gy	SRB_FBL-03G_200µg	SRB_FBL-03G_200µg_6Gy	SRB_FBL-03G_300µg	SRB_FBL-03G_300µg_6Gy
14	-1							
17	-1							
20	-1							
20	-1							
24	-1							
24	-1							
27	-1							
27	-1							
27	-1							
27	-1							
17		-1						
20		-1						
24		-1						
27		-1						
29		-1						
31		-1						
31		-1						
20		-1						
31		-1						
31		-1						
20			-1					
24			-1					
27			-1					
31			-1					
31			-1					
40			0					
40			0					
40			0					
40			0					
40			0					
20				-1				
20				-1				
27				-1				
40				0				
40				0				
40				0				
40				0				
40				0				
40				0				
40				0				
20					-1			
27					-1			
31					-1			
40					0			
40					0			
40					0			
40					0			
40					0			

40					0			
40					0			
20						-1		
27						-1		
40						0		
40						0		
40						0		
40						0		
40						0		
40						0		
40						0		
40						0		
20							-1	
27							-1	
40							0	
40							0	
40							0	
40							0	
40							0	
40							0	
40							0	
40							0	
40								0
40								0
40								0
31								-1
40								0
40								0
40								0
40								0
40								0
40								0

Table 2. The raw mice survival data shown above corresponds to Figure 5C. The negative values are indicative of when mice reach the endpoint of the study.
