# Supplementary figures and images for: Corrigendum: Flavonoid Derivative of Cannabis Demonstrates Therapeutic Potential in Preclinical Models of Metastatic Pancreatic Cancer
Source: Front Oncol. 2020 Aug 21;10:1434. doi: 10.3389/fonc.2020.01434 (PMC7472526; doi:10.3389/fonc.2020.01434)

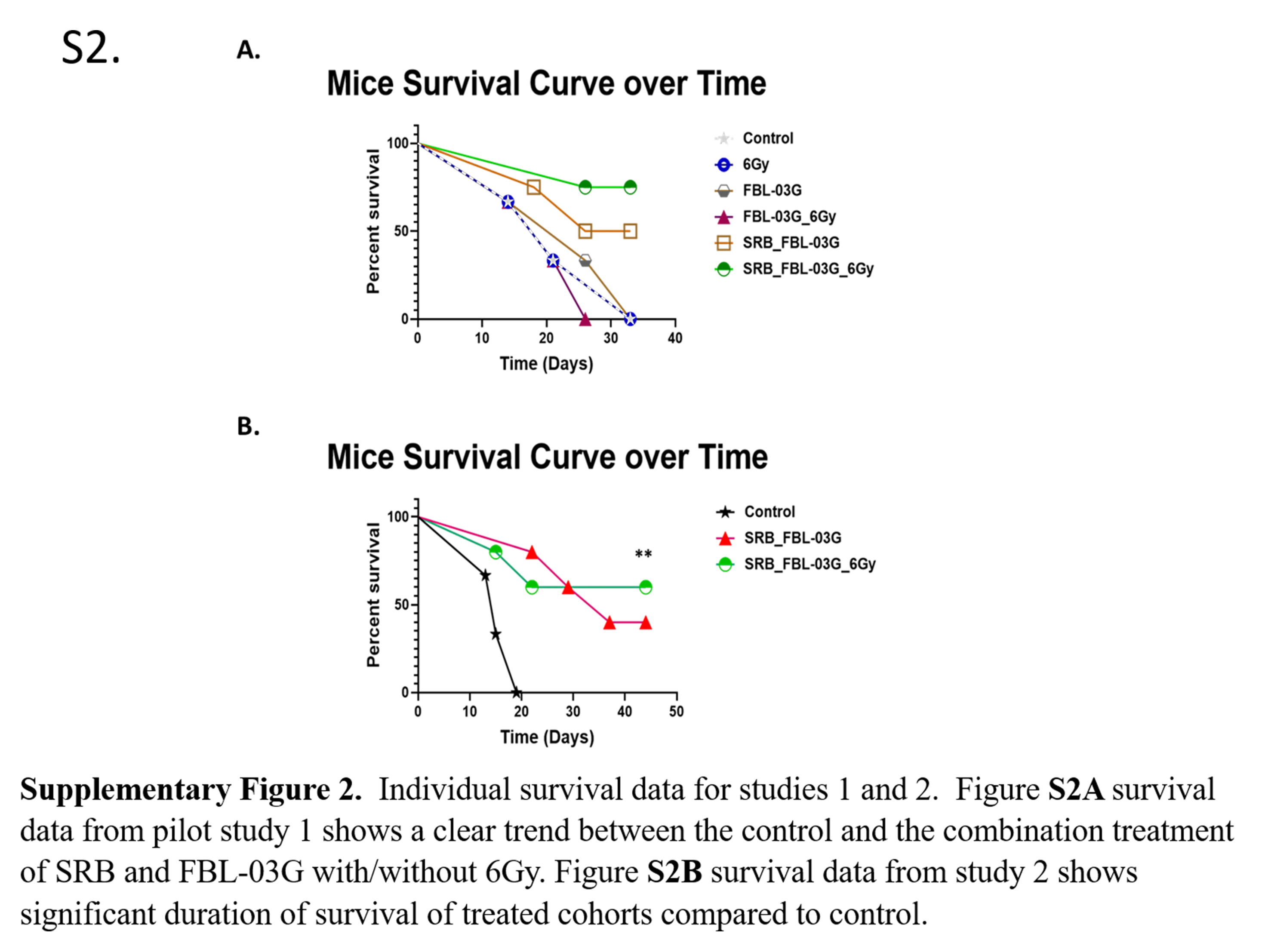

Supplement: Supplementary file 1 [file Image_1.PNG]
